# The liminal position of Nuclear Physics: from hadrons to neutron stars

**DOI:** 10.1098/rsta.2023.0128

**Published:** 2024-07-23

**Authors:** Marina Petri, Alexandros Gezerlis

**Affiliations:** ^1^ School of Physics, Engineering and Technology, University of York, York YO10 5DD, UK; ^2^ Department of Physics, University of Guelph, Guelph, Ontario N1G 2W1, Canada

**Keywords:** nuclear physics, hadron physics, neutrino physics, nuclear structure, nuclear reactions, nuclear astrophysics

The atomic nucleus was discovered more than a century ago, yet an array of foundational questions remains unanswered regarding both the force that keeps its constituents together and how one builds up heavier systems. In recent decades, other exciting frontiers have been reached, e.g. exploring new or invisible particles (neutrinos, dark matter). Nuclear physics plays a central role in our understanding of both the atomic nucleus and seemingly unrelated things, e.g. our understanding of the interaction of neutrinos with matter, or how stars are born and die. With this theme issue, we are bringing together contributions from prominent researchers in the field in order to showcase the vibrancy of nuclear physics, covering a broad selection of current frontier topics. Central to this theme issue is the interplay between experiment and theory as the driving force of breakthroughs in the field. With advances in both nuclear theory and experimental capabilities, it is more important than ever to build the foundations of a truly collaborative community. It is only when experiments are informed by theory, and theory guided by experiments, that real breakthroughs can be achieved. This theme issue will showcase these synergies, inspiring and triggering further such collaborations.

This special issue brings together the wider nuclear physics community, from traditional nuclear structure, reactions and dynamics, to hadron physics, nuclear astrophysics and neutrino physics. Some of the contributions venture further afield and touch upon the relevance of machine-learning techniques to nuclear physics, while others discuss strongly correlated matter that (while sharing many commonalities with neutron matter) is experimentally accessible using ultracold atoms (not nucleons). This reinforces the liminal position of nuclear physics across many research frontiers. We also hope that this issue will prompt new theory-experiment synergies and that it will plant the seeds for cross-collaborations amongst different fields.

All contributions to this theme issue involve a mixture of theoretical and experimental work: exactly because nuclear physics stands at the interface between different scales, research in the field needs to make use of all available tools, both traditional/established and novel/exploratory. To make matters concrete, in what follows we will briefly discuss the contributions that now lie between one set of covers, starting with the mainly theoretical ones. In addition to briefly highlighting what is novel in the works contained herein, we also attempt to find a guiding thread through all of the original works that form the present theme issue.

The work by Soma and Duguet nicely illustrates the aforementioned juxtaposition of traditional and novel approaches. The article hones in on the tool known as effective single-particle energies, which originally arose in the context of single-particle studies of shell structure in atomic nuclei. The authors clearly delineate the non-observable character of effective single-particle energies but do not limit themselves to criticism: they make contact with both modern *ab initio* quantum many-body studies of nuclei and experimental work. Specifically, they employ the successful Gorkov–Green’s function approach to tackle a system recently studied experimentally at iThemba LABS. Through a combination of pedagogical exposition and original results, the scheme-dependence of effective single-particle energies is brought into sharp relief. While results focus on a specific neutron spin-orbit splitting along *N* = 20 isotones, the lessons gleaned from such studies are much more general.

The next theoretical work (as well as the two following ones) falls into the large category of Monte Carlo approaches, employing random numbers to study a problem in microphysics. Specifically, Drissi *et al.* employ a continuum quantum Monte Carlo approach: variational Monte Carlo, in which a sophisticated guess for the ground-state wave function of a quantum many-body system is optimized in order to estimate physical properties as accurately as possible. A promising such guess that has been heavily used recently is that of neural-network quantum states; the authors go over neural wave functions with an added emphasis on the optimization algorithm. They discuss the Kronecker Factored Approximate Curvature optimizer in detail and transcend its shortcomings via novel extensions. The authors also take the opportunity to reformulate their approach using the language of game theory; via the concept of decision geometry, they propose a new optimizer that behaves better than standard ones (or the authors’ own earlier approaches). While the physical system tackled involves a central interaction, such approaches hold the promise of improving future nuclear calculations of related quantities.

The work by Morrell *et al.* also studies spin-1/2 fermions (i.e. does not specifically address nucleons), which could function as a foundation for later nuclear studies. The main tool employed here is also quantum Monte Carlo; unlike the earlier work that used a continuum approach, Morrell *et al.* place their strongly interacting particles on a spatial lattice in addition to discretizing the (imaginary) time evolution. Crucially, the authors of this work are able to use very few temporal steps/a very coarse grid in the time direction via a quantum cumulant expansion, i.e. using automated algebra rather than the more typical numerical approach. The system studied is not just a toy problem: cold fermions at unitarity have been extensively probed in lab work over the last two decades using dilute atomic systems. Thus, the authors provide specific comparisons with results from an MIT experiment, not only for the pressure, but also for the compressibility and heat capacity. Unlike much of nuclear-structure physics, this work is carried out at finite temperatures; such approaches to the thermodynamics of strongly coupled systems can guide future work on nuclear astrophysics.

The work by Curry *et al.* similarly employs a lattice Monte Carlo formalism, this time at 0 temperature. The specific technique used is auxiliary-field quantum Monte Carlo, which has in the past been quite successful in condensed-matter and cold-atom studies; its main characteristic feature is the choice of working in a Slater-determinant space. The authors start by benchmarking against one- and two-dimensional results for the Hubbard model, a mainstay of solid-state physics. They then discuss experimentally relevant results for cold fermions at unitarity (also touched upon in the previous paragraph), paying close attention to the question of how to extrapolate away the lattice/discretization effects. The authors then turn to nuclear systems, carefully tuning the two-body lattice interaction such that it reproduces the neutron–neutron scattering length and effective range. They round things off by studying the energy of a few to many strongly interacting neutrons for a variety of lattice sizes. Given the power of the many-body technique and the promise of these neutron-matter results, one can expect extensions to bound states/atomic nuclei using similar approaches in the near future.

Moving to a more synergistic contribution on both experiment and theory, Roth and Petri review the developments of the *ab initio* frontier, where chiral effective field theories of the strong interaction are being exploited to derive nuclear interactions and form the foundation of modern *ab initio* methods. Such an approach is bringing us closer to the understanding of nuclei from first principles, rooted in the theory of the strong interaction, quantum chromodynamics (QCD). Key to advancing the frontiers of *ab initio* theory is pioneering experiments on atomic nuclei that confront *ab initio* predictions and provide unparalleled benchmarks for the development and refinement of the theoretical approaches. This contribution puts forward selected experimental efforts along the carbon and oxygen chains that aim at validating these calculations, with a focus on electromagnetic observables, which present a particular challenge for *ab initio* theory. What will decisively drive future developments and breakthroughs in our understanding of the atomic nucleus from first principles is the strong synergy between experiment and theory and this contribution celebrates this.

The work of Uesaka and Itagaki discusses the manifestation of non-uniformity in nuclei in the form of nuclear clustering. This review presents an interesting collection of examples of nuclear clustering throughout the nuclear chart and considers the underlying driving mechanism for this phenomenon, in terms of the tensor and spin-orbit forces as well as modern nucleon–nucleon interactions. Clustering plays a critical role in the origin of elements with further implications for nuclear fission and the potential to produce superheavy elements. Experimental breakthroughs in probing clustering in medium and heavy nuclei are explored and new experimental approaches, through knockout reactions, are presented.

Continuing the theme of synergistic theoretical and experimental work, and extending the impact of nuclear physics to the cosmos, Wiedeking and Goriely discuss the impact of photon strength functions (PSF) and nuclear level densities (NLD) on shaping the outcomes of various nucleosynthetic processes. The authors give an overview of both experimental and theoretical methods to determine PSF and NLD, how they impact the important quantities that enter the astrophysical simulations, i.e. the reaction rates, and subsequently, how this affects the obtained abundances. Indeed, large uncertainties for (*p*,*γ*), (*n*,*γ*) and (*α*,*γ*) reaction rates across many regions of the nuclear chart are uncovered from diverse NLD and PSF model combinations, leading to potentially significant abundance variations of the nucleosynthesis processes. This contribution reflects on the critical role that nuclear physics plays in the cosmos and highlights how advances in theory and experimentation are enhancing our grasp of the origin of elements.

Nuclear physics input is critical not only to our understanding of the origin of the elements but to other exotic objects in the cosmos, most notably neutron stars. Neutron stars, dense remnants of supernova explosions, contain matter that reaches extreme densities and pressure. Currently, their true nature remains an open question. The presence of hyperons, particles containing strange quarks, affects the equation of state, influencing the neutron star’s size and internal structure. Understanding the behaviour of hadrons and hyperons within neutron stars sheds light on the fundamental physics governing these enigmatic celestial objects. The excitation spectra of hyperons provide critical constraints to models of big-bang nucleosynthesis with the majority of visible matter in the Universe progressing through excited hyperon states. Novel experimental efforts that allow us to bridge the knowledge gap on the interaction between hyperons and nucleons and underpin the rich hyperon excitation spectra are underway at the new K-long facility. Zachariou *et al.* discuss the K-Long facility at the Thomas Jefferson Laboratory and its potential to investigate the hyperon–nucleon interaction and hyperon spectroscopy. Such experimental constraints are critical for understanding and testing QCD in the strange sector and for underpinning our understanding of dense nuclear matter.

Scientific technological advances have allowed us to discover new phenomena, e.g. neutrino oscillations. Although this type of experiment lies well within the domain of high-energy physics, nuclear physics has a fundamental role to play, e.g. in measurements of single and double beta-decay spectra that illuminate the scale and nature of the neutrino mass, and in neutrino–nucleus scattering that underpins oscillation experiments. It is these experimental advances that have brought down barriers between long-standing traditional fields of physics. The contribution by Parno *et al*. celebrates the intersection between long-standing traditional fields of physics by discussing experimental neutrino physics in a nuclear landscape. The authors present two nuclear laboratories for exploring neutrino physics, through nuclear beta decays, to shed light on the absolute neutrino-mass scale and answer the question of whether neutrinos are Majorana particles. Parno *et al.* further discuss the nuclear physics of high-energy neutrino interactions, essential for interpreting long-baseline neutrino-oscillation experiments, the use of low-energy neutrino scattering to illuminate nuclear properties and supernova nucleosynthesis, neutrino probes of fission reactors, searches for sterile neutrinos and applications in quantum sensing.

This issue concludes with one of the most exciting facilities for nuclear physics research that is coming online in the near future, the Facility for Antiproton and Ion Research (FAIR), and discusses Nuclear Structure opportunities with GeV radioactive beams across a range of fields and scales: from low-energy physics via the investigation of multi-neutron systems and halos, to high-density nuclear matter and the equation of state, following heavy-ion collisions, fission, study of short-range correlations in nuclei, as well as hypernuclei. The R^3^B setup features prominently as a versatile experiment to perform kinematically complete measurements with high-energy radioactive ion beams, and various physics cases pursued within its experimental capabilities are presented. Even more interestingly, the authors put forward future opportunities at FAIR of studying exotic nuclei scattering off light ions and electrons in storage rings, as well as the concept of multi-GeV experiments with radioactive beams.

As our brief summaries show, the works presented here involve many length scales, subfields and methodologies. It is worth reiterating that bringing together both theoretical and experimental works in the same venue (while not commonly done) is a good way to highlight past progress and jumpstart future discoveries. We have certainly enjoyed working with the authors, referees and the editorial office in putting this theme issue together. We hope the reader will, too.

## Data Availability

This article has no additional data.

